# Interactive effects of leg autotomy and incline on locomotor performance and kinematics of the cellar spider, *Pholcus manueli*

**DOI:** 10.1002/ece3.3231

**Published:** 2017-07-21

**Authors:** Gary W. Gerald, Moriah M. Thompson, Todd D. Levine, Kerri M. Wrinn

**Affiliations:** ^1^ Biology Department Nebraska Wesleyan University Lincoln NE USA; ^2^ Department of Life Sciences Carroll University Waukesha WI USA; ^3^ Department of Biological Sciences University of Wisconsin‐Rock County Janesville WI USA

**Keywords:** climbing, duty factor, performance, speed, stride cycle time, stride length

## Abstract

Leg autotomy can be a very effective strategy for escaping a predation attempt in many animals. In spiders, autotomy can be very common (5–40% of individuals can be missing legs) and has been shown to reduce locomotor speeds, which, in turn, can reduce the ability to find food, mates, and suitable habitat. Previous work on spiders has focused mostly on the influence of limb loss on horizontal movements. However, limb loss can have differential effects on locomotion on the nonhorizontal substrates often utilized by many species of spiders. We examined the effects of leg autotomy on maximal speed and kinematics while moving on horizontal, 45° inclines, and vertical (90°) inclines in the cellar spider *Pholcus manueli*, a widespread species that is a denizen of both natural and anthropogenic, three‐dimensional microhabitats, which frequently exhibits autotomy in nature. Maximal speeds and kinematic variables were measured in all spiders, which were run on all three experimental inclines twice. First, all spiders were run at all inclines prior to autotomization. Second, half of the spiders had one of the front legs removed, while the other half was left intact before all individuals were run a second time on all inclines. Speeds decreased with increasing incline and following autotomy at all inclines. Autotomized spiders exhibited a larger decrease in speed when moving horizontally compared to on inclines. Stride length decreased at 90° but not after autotomy. Stride cycle time and duty factor increased after autotomy, but not when moving uphill. Results show that both incline and leg autotomy reduce speed with differential effects on kinematics with increasing incline reducing stride length, but not stride cycle time or duty factor, and vice versa for leg autotomy. The lack of a significant influence on a kinematic variable could be evidence for partial compensation to mitigate speed reduction.

## 1 INTRODUCTION

Autotomy, self‐amputation of an appendage, is a common adaptation observed in a variety of animal taxa (e.g., lizards, salamanders, crustaceans, insects, and spiders) that should be selected for if it increases the chance of surviving a predatory encounter (Edmunds, 1974). Despite the obvious benefit of immediate survival, limb autotomy may also have severe negative consequences on locomotor performance, which is crucial for finding food, mates, and suitable habitat (Arnold, 1983). In spiders, limb loss has been shown to hinder sprint speeds (Amaya, Klawinski, & Formanowicz, 2001; Apontes & Brown, 2005) and can affect the ability of spiders to capture prey items (Wrinn & Uetz, 2008). Other costs associated with limb loss in spiders include increased vulnerability of males during aggressive interactions with females (e.g., Taylor & Jackson, 2003) and decreased male mating success (Uetz, McClintock, Miller, Smith, & Cook, 1996). Despite the potentially high cost of limb loss, spiders will readily autotomize legs during encounters with predators and aggressive conspecifics (Formanowicz, 1990; Klawinski & Formanowicz, 1994; Punzo, 1997) and rates of reduced limb numbers are relatively high in some spider species, varying from 5 to 40% (Dodson & Beck, 1993; Foelix, 1996; Johnson & Jakob, 1999; Punzo, 1997).

Context is crucial in understanding the costs of autotomy on individual animals (Wrinn & Uetz, 2008). This is especially true during locomotion as it may be context dependent in spiders (Pruitt & Husak, 2010). Although reductions in sprint speeds have been found in a few species of arachnids following leg autotomy (e.g., Amaya et al., 2001; Apontes & Brown, 2005; Houghton, Townsend, & Proud, 2011), these studies found reductions in speed during horizontal locomotion. Arguably, examining movements on level substrates exclusively could underestimate potential locomotor costs in nature because many spiders must travel up and down many types of three‐dimensional, inclined substrates and within webs, as they use preferred microhabitats (Prenter, Fanson, & Taylor, 2012). Hence, examining movement in more ecologically relevant contexts (e.g., on inclines) could provide a better understanding of locomotor costs and any compensatory mechanisms that might be present in spiders to counteract leg autotomy. Locomotion up and down inclines has been shown to be more energetically expensive in other animals (Full & Tullis, 1990; Jayne & Irschick, 1999; Lipp, Wolf, & Lehmann, 2005). Previous studies comparing vertical climbing and horizontal running in spiders have found that most spiders exhibit a reduction in performance during vertical climbing and that smaller spiders (usually males) are less impaired during climbing (Brandt & Andrade, 2007; Prenter, Perez‐Staples, & Taylor, 2010a; Prenter et al., 2012). No study, to our knowledge, has examined the potential costs of leg autotomy in spiders on incline locomotion. An assessment of the locomotor costs of leg autotomy on both level and nonlevel substrates is crucial because spiders commonly traverse surfaces that range from flat (0°) to vertical (90°) and even upside down, while exhibiting high rates of leg loss.

In this study, we examined the effects of inclined surfaces and leg autotomy on the locomotor performance of the cellar spider, *Pholcus manueli* Gertsch, 1937. This is a ubiquitous, invasive species commonly found in basements and outbuildings in the Midwest and northeastern U.S.A. Although abundant, little is known of their natural history. However, pholcid spiders commonly autotomize legs but do not exhibit leg regeneration (Uhl, 1998). We also measured kinematic variables to quantify how leg loss impacts stride mechanics on different inclined surfaces. We hypothesized that leg autotomy will hinder locomotor performance and alter kinematics and that these effects will be exacerbated as incline increases.

## 2 MATERIALS AND METHODS

### 2.1 Study species and autotomization

Forty‐three penultimate and adult *Pholcus manueli* were collected in June 2015 from an outbuilding in Lincoln, Nebraska, U.S.A (40°49′49″N; 96°37′18″W). Spiders were maintained individually in plastic containers (volume = 600 ml) and fed 3–5 fruit flies 2 times per week and provided water ad libitum. On feeding days, trials were conducted prior to feeding. Initial locomotor trials were conducted 24–48 hr following capture. Total body mass was measured before and after autotomization treatments. Spiders were autotomized by grabbing and holding the left or right front leg with forceps until autotomy occurred. Half of the autotomized spiders had the left front leg removed, while the other half had the right leg removed. The first spider selected for autotomization was assigned to have the left leg removed based on a coin flip. We alternated removing right and left front legs for subsequent spiders.

### 2.2 Locomotor trials

Using a repeated‐measures design, all spiders were placed randomly into either the control or autotomy treatment group. First, all spiders (from both treatments) were run on one horizontal (0°) surface and uphill on two nonhorizontal (45° and 90° inclines) surfaces. Second, legs were removed from spiders assigned to the autotomy treatment as previously mentioned. Spiders in the control group were handled similarly, but without removing a leg. Lastly, all spiders (from both treatments) were run again on all aforementioned inclines (0°, 45°, and 90°). The sequence of inclines for the first run varied, and individuals were randomized among three sequences: 45°–0°–90°; 0°–90°–45°; 90°–0°–45°. The sequence was kept the same for spiders of both treatments during the second run: 45°–0°–90°.

All locomotor trials were conducted on a 1‐m long‐track using a styrofoam substrate marked at 5‐cm intervals and set so that it was either 0°, 45° or 90° from the plane of the horizontal surface. During trials, spiders were encouraged to move at maximal speed by following them with a finger and tapping the substrate gently behind them similarly for all individuals. Spiders were encouraged to begin moving at maximal speed 3–5 cm prior to entering the portion of the substrate used to time individual trials. Each individual was encouraged to move at least three successive times within the same trial, and the maximal speed was used for all subsequent analyses. This was done separately for each condition in the experiment. In other words, each spider was videotaped running during six conditions (each of the three inclines x before and after autotomy treatment) over the course of this project. Spiders were given at least 24 hr to rest between being run in each condition. Therefore, locomotor speeds were assessed everyday for 6 days per individual.

All trials were videorecorded at 300 frames per second with a digital camcorder (Casio Exilim Pro EX‐F1). From videos, we recorded maximal speed (cm/s), stride length (cm), stride cycle time (s), and duty factor. Speed was determined by measuring the time it took to travel several 5‐cm increments. The fastest was used for all speed analyses and for quantification of kinematic variables of all legs over that locomotor bout. Stride length was defined as the distance between the placement of one leg onto the substrate to the subsequent placement of the same leg. Stride cycle time was the time that it took to complete one stride length. Duty factor was defined as the percentage of the stride cycle time during which a particular leg was in contact with the ground.

### 2.3 Statistical analyses

Using intact spiders, the effects of incline on speed and kinematic variables were examined using ANCOVAs with body mass as a covariate. The effects of autotomy treatment and the interaction between autotomy treatment and incline on speed and kinematic variables were analyzed using linear mixed‐effects models (LMEs) with autotomy treatment and incline as fixed effects and individual as a random effect. The mixed models used the difference in speed and kinematics between successive runs (second minus first runs) as dependent variables. In these models, we assessed the interactions between autotomy treatment and incline to determine if autotomy influenced the dependent variables differently on different inclines. The influence of leg position was left out of the aforementioned models because no significant differences were detected among legs for any kinematic variable during horizontal movements. Consequently, statistical analyses on all kinematic variables were conducted using the second pair of remaining legs for both intact and autotomized individuals. All statistical analyses were conducted using XLSTAT (version 2015.3.01).

## 3 RESULTS

### 3.1 Speed

**Figure 1:**
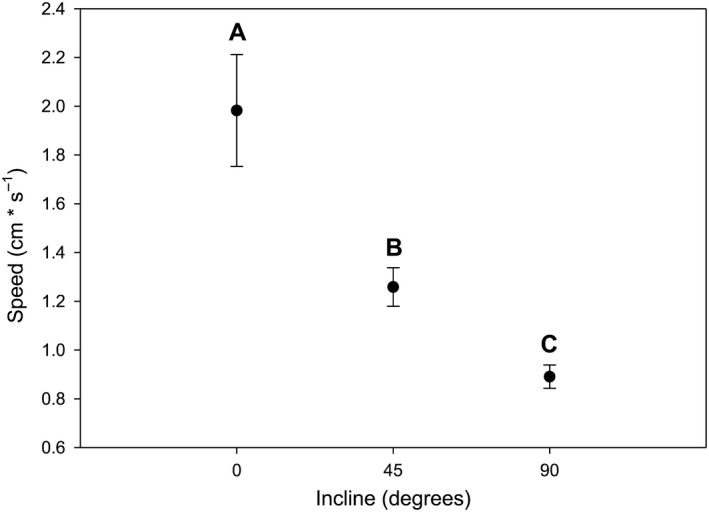
Influence of incline (0°, 45°, and 90°) on uphill maximal speed of cellar spiders (*Pholcus manueli*) before leg autotomy (*N* = 43). Error bars represent ± SE. Different letters denote statistical differences at the 0.05 level

**Figure 2:**
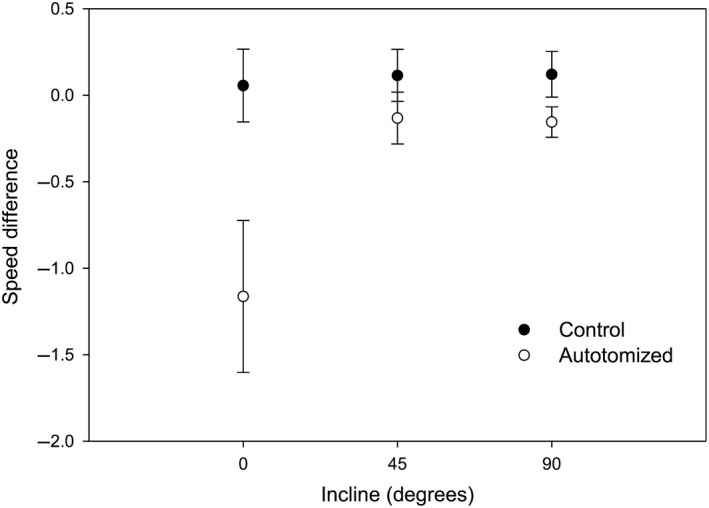
The influence of incline (0°, 45°, and 90°) on the difference in maximal speed between the second and first runs of cellar spiders (*Pholcus manueli*) assigned to the control (nonautotomized) and autotomized groups. Overall, autotomized spiders exhibited a larger decrease in speed on all inclines. The larger decrease in speed following autotomization observed on the 0° incline was statistically significant (*p *=* *.04) compared to the other inclines. Error bars represent ± SE

Overall, maximal speeds decreased with increasing incline for all spiders in the study (Figure 1; ANCOVA, *F*_2,128_ = 10.01, *p *<* *.0001). There was no significant effect of mass on speed (ANCOVA; *F*_1,128_ = 0.42, *p *=* *.52). Autotomized spiders exhibited a decrease in speed compared to control spiders across all inclines (Figure 2; LME, *F*_1,82_ = 8.25, *p *=* *.005). A significant interaction between incline and autotomy treatment was found (LME, *F*_2,82_ = 3.24, *p *=* *.044) that was due to the larger decrease in speed on 0° inclines following autotomy compared to 45° and 90° owing to the initial faster speeds on 0° inclines (Figure 2).

### 3.2 Stride length

**Figure 3:**
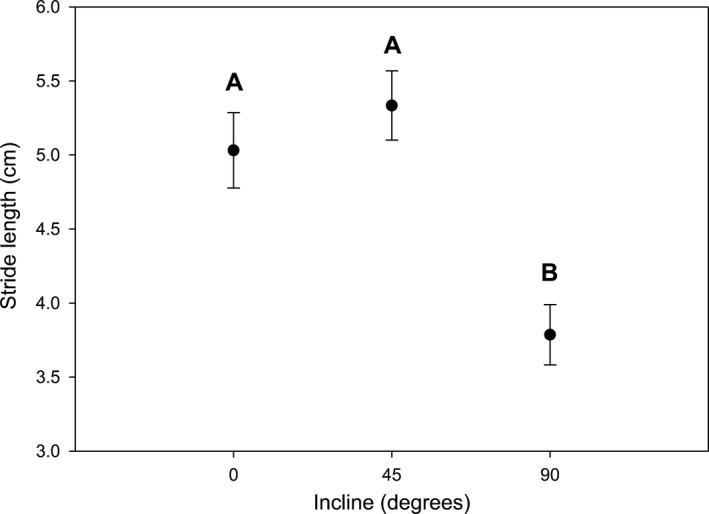
Influence of incline (0°, 45°, and 90°) on stride lengths of cellar spiders (*Pholcus manueli*) before leg autotomy (*N* = 43). Error bars represent ± SE. Different letters denote statistical differences at the 0.05 level

**Figure 4:**
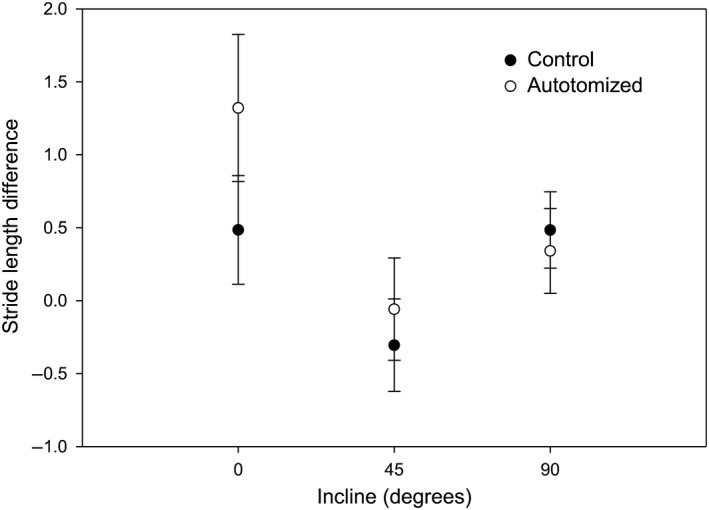
The influence of incline (0°, 45°, and 90°) on the difference in stride length between the second and first runs of cellar spiders (*Pholcus manueli*) assigned to the control (nonautotomized) and autotomized groups. There was no significant difference between control and autotomized spiders (*p *=* *.31). However, differences at 45° were significantly lower than 0° and 90° (*p *=* *.01). Error bars represent ± SE

Stride length was significantly influenced by changes in incline (Figure 3; ANCOVA, *F*_2,128_ = 20.50, *p *<* *.0001). Stride length during movement at 90° was lower than that of 0° and 45°. There was no significant effect of body mass on stride length (ANCOVA, *F*_1,128_ = 1.07, *p *=* *.30). There were no significant effects of autotomy treatment (Figure 4; LME, *F*_1,82_ = 1.03, *p *=* *.314) or autotomy treatment/incline interaction (LME, *F*_2,82_ = 0.98, *p *=* *.381) on stride length. However, there was a significant incline effect with the difference in stride length at 45° being smaller than at 0° and 90°, which were above zero showing that stride length increased between the first and second trials at 0° and 90° (Figure 4; LME, *F*_2,82_ = 4.65, *p *=* *.012).

### 3.3 Stride cycle time

**Figure 5:**
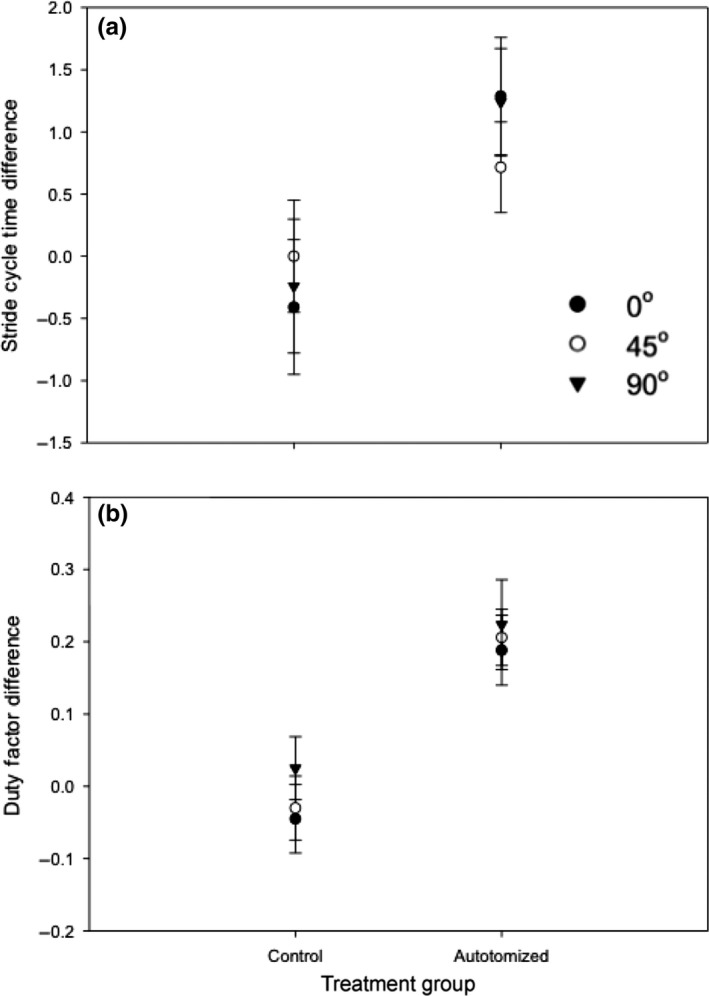
The influence of incline (0°, 45°, and 90°) on the difference in stride cycle time (a) and difference in duty factor (b) between the second and first runs of cellar spiders (*Pholcus manueli*) assigned to the control (nonautotomized) and autotomized groups. Although there was no influence of incline, there was a treatment effect (*p *<* *.001) with autotomized spiders exhibiting a greater increase in both stride cycle time and duty factor. Error bars represent ± SE

There was no significant influence of incline (ANCOVA, *F*_2,128_ = 2.12, *p *=* *.102) or body mass (ANCOVA, *F*_1,128_ = 1.43, *p *=* *.234) on stride cycle time. Autotomy treatment resulted in an increase in stride cycle time across all inclines (Figure 5a; LME, *F*_1,82_ = 10.35, *p *=* *.002). There was no significant effect of incline (LME, *F*_2,82_ = 0.12, *p *=* *.890) or autotomy treatment/incline interaction (LME, *F*_2,82_ = 1.92, *p *=* *.153) on the difference in stride cycle times.

### 3.4 Duty factor

There was no significant effect of incline (ANCOVA, *F*_2,128_ = 1.06, *p *=* *.370) or body mass (ANCOVA, *F*_1,128_ = 1.78, *p *=* *.184) on duty factor. Autotomy treatment resulted in an increase in duty factor across all inclines (Figure 5b; LME, *F*_1,82_ = 21.43, *p *<* *.001). There was no significant influence on incline (LME, *F*_2,82_ = 1.07, *p *=* *.348) or autotomy treatment/incline interaction (LME, *F*_2,82_ = 1.86, *p *=* *.162) on the difference in duty factor.

## 4 DISCUSSION

As predicted, we found that speeds of *P. manueli* decreased with increasing incline. A reduction in performance on sloped surfaces has been seen in other animals (reviewed in Birn‐Jeffrey & Higham, 2014). This decrease in performance is likely due to the increased muscular work needed to move an animal's center of mass against the increasing effects of gravity. Previous studies on limbed vertebrates, such as lizards, have found that increasing inclines reduce performance and increase energetic costs in a size‐dependent way with larger body sizes being more hindered on steeper slopes (Farley & Emshwiller, 1996; Huey & Hertz, 1982). In spiders, the gravity hypothesis proposes that selection for increased speed during vertical climbing in males selects for smaller body sizes because of the aforementioned reduction in speed in larger individuals during inclined movement (Moya‐Larano, Halaj, & Wise, 2002). This hypothesis is supported by studies that have shown that smaller spiders usually climb faster than larger ones (Moya‐Larano, Vinkovic, Allard, & Foellmer, 2007). However, when comparing horizontal running to vertical climbing in spiders, results can be mixed with some species climbing faster than they run, while others display the reverse (Prenter, Perez‐Staples, & Taylor, 2010b).

At intermediate, nonvertical inclines, burst speeds have been found to be lower in a jumping spider (*Jacksonoides queenslandica*) and an orb‐web spider (*Nephilia plumipes*; Prenter et al., 2012). However, there appears to be no interaction between locomotor hindrance on steeper inclines and body size, despite the sexual dimorphism present in many spiders (Prenter et al., 2010a, 2012). Pholcid spiders inhabit corners of sheltered habitats, such as caves, basements, and other areas with significant vertical and overhanging horizontal structures. Although pholcids are likely to have evolved in such spaces, they must still overcome the biomechanical limitations imposed by gravity observed in other animals.

Our hypothesis, that both incline and leg autotomy would reduce maximal speeds via differential changes to stride kinematics, was supported. This aligns with previous work on wolf spiders (Lycosidae) that found that autotomized spiders were slower following leg autotomy during horizontal running (Amaya et al., 2001; Apontes & Brown, 2005). Similar reductions in locomotor performance resulting from loss of a limb have been shown in stick insects (Carlburg, 1984), harvestman (Dominguez et al., 2016; Guffey, 1999; Houghton et al., 2011), decapod crustaceans (Juanes & Smith, 1995), and sea stars (Bingham, Burr, & Wounded Head, 2000). Additionally, Fleming and Bateman (2007) found that leg autotomy reduced endurance and increased the energetic cost of locomotion in field crickets (*Gryllus bimaculatus*).

Ours is the first study to show that the locomotor hindrance following leg autotomization is reduced when moving uphill (Fig. 2). The smaller differences in speed between intact and autotomized spiders on inclines are likely due to one of three reasons that are not mutually exclusive. First, the reduction in speed due to autotomy on inclines might be overshadowed by the concomitant decrease in speed resulting from incline‐dependent limitations. Secondly, spiders may be able to more easily compensate for a lost leg when moving more slowly uphill. These hypotheses could be tested by examining kinematic changes during maximal and submaximal speeds during horizontal movements before and after leg autotomy. Lastly, we cannot rule out that speeds did not increase between the first and second sets of trials (24‐hr difference) due to spiders adjusting to moving with one less leg over time or learning associated with running on the same track. If performance did slightly improve between the first and second trials, the speed differences we observed (Fig. 2) would be underestimates. Future studies that measure performance at different times following autotomy would be helpful in determining how spiders mitigate the effect of leg loss on locomotion and other behaviors.

We also found that both intact and autotomized spiders exhibited reduced stride lengths at the steepest experimental incline. A reduction in stride length with incline has been noted in humans (Slowinski et al., 2008), lizards (e.g., Irschick & Jayne, 1998; Jayne & Irschick, 1999), and in fiddler crabs (Gerald & Thiesen, 2014). In spiders, a loss of a leg does disrupt the diagonal sequence of footfalls characteristic of eight‐legged locomotion in arachnids (Foelix, 1996). However, Wilson (1967) found that tarantulas increase stride length to fill the gap of a missing leg to mitigate the reduction in speed. Similarly, Escalante, Albin, and Aisenberg (2013) observed an increase in stride length in harvestman (*Holmbergiana weyenberghi*) that were missing at least one leg. Although due mostly to weight loading, a similar compensation (i.e., increase in stride length on inclines) was observed in male fiddler crabs (*Uca pugilator*) following removal of the enlarged claw (Gerald & Thiesen, 2014). However, we found no significant increase in stride length following autotomization on any experimental incline. It is possible that the lack of a significant decrease in stride length could represent a partial kinematic compensation as stride cycle times increased on all inclines after leg removal. In other words, the effort to maintain stride lengths despite the disruption in the step sequence resulting from leg loss could mitigate the decrease in speed.

We found that stride cycle time, which is directly related to stride frequency, and duty factor, the proportion of the stride in which the leg is in contact with the substrate, increased after leg autotomy, but not with increasing incline. This shows that the decreases observed in maximal speed after leg autotomy are due primarily to the increase in stride cycle times and duty factor and not a decrease in stride length. The slower strides could result from the need to utilize an altered step sequence following leg loss. The lack of an incline effect suggests compensation for the reduced speed by maintaining stride frequency when transitioning from horizontal to inclined movements. Previous studies on horses (Williams, Nankervis, Colberne, Marlin, & Schroter, 2009) and lizards (Foster & Higham, 2012) have shown that duty factor increases with incline. As seen in other limbed animals, the increase in duty factor observed in autotomized spiders is directly related to the decrease in speed (Alexander, 1989). Although increases in duty factor provide more time for ground contact needed to generate ground reaction forces (Pontzer, 2005), higher duty factors are related to slower strides. After autotomy, in addition to increasing overall duty factor for all legs, differences in duty factor among legs were not detected suggesting the relative contribution of the intermediate legs to force generation increases to compensate for a lost leg, regardless of incline. More kinematic studies on spiders are needed to tease apart the plasticity in stride length, stride cycle times, duty factors, and ground reaction forces among the different legs on inclines and before and after autotomy.

In conclusion, we found that both incline and leg autotomy hinder locomotor speeds but affect stride kinematics of pholcid spiders differently. Spiders experienced reduced speeds when moving up the steepest inclines because of a decrease in stride length. However, leg autotomy resulted in a decrease in their maximal speeds on all inclines via an increase in stride cycle time and duty factor. Interestingly, stride length was not affected by leg autotomy, suggesting some mitigation in speed reduction via the effort to maintain typical stride lengths. We acknowledge that one must be cautious to generalize these results to all arachnids for several reasons. First, this study examined the removal of one of the first pair of legs only. The diagonal rhythm of the stepping patterns of spiders differs among alternating pairs of legs, resulting in differential contributions to overall force production among legs (Biancardi, Fabrica, Polero, Loss, & Minetti, 2011). For example, the fourth pair of legs has been suggested to supply the most force to propel the spider relative to the other legs (Biancardi et al., 2011). Utilizing the same inclines, the removal of any of the other legs could result in different kinematic alterations and, thus, disparate influences on performance. Hence, studies examining the removal of other legs or more than one leg would be especially helpful in generalizing the effects of leg autotomy on spider locomotion. Secondly, this study used a species that is specialized for traversing up and down three‐dimensional, aboveground structures in nature as opposed to a more ground‐dwelling species, such as wolf spiders. Although species in the genus *Pholcus* spend much time in webs, it must also traverse substrates outside the web when searching for new foraging areas or mates, during aggressive behaviors with conspecifics, or during predator avoidance (Hutton & Rypstra, 2016; Uhl, 1998). In order to properly examine autotomy effects in ecologically relevant contexts, a larger number of species with varying morphology, behaviors, and ecological specializations both on and off webs should be investigated.

## DATA ACCESSIBILITY

Data will be deposited into DRYAD.

## CONFLICT OF INTEREST

The authors declare no conflict of interests.
